# Observations of Cherenkov‐Like Radial Wake in Water Waves

**DOI:** 10.1002/advs.202412638

**Published:** 2025-01-28

**Authors:** Zijian Qin, Lian Shen, Yihao Yang, Hongsheng Chen, Huaping Wang

**Affiliations:** ^1^ Key Laboratory of Ocean Observation‑Imaging Testbed of Zhejiang Province Ocean College Zhejiang University Hangzhou 310058 China; ^2^ State Key Laboratory of Extreme Photonics and Instrumentation ZJU‐Hangzhou Global Scientific and Technological Innovation Center Zhejiang University Hangzhou 310027 China; ^3^ Interdisciplinary Center for Quantum Information State Key Laboratory of Modern Optical Instrumentation College of Information Science and Electronic Engineering Zhejiang University Hangzhou 310027 China; ^4^ Key Lab. of Advanced Micro/Nano Electronic Devices & Smart Systems of Zhejiang Jinhua Institute of Zhejiang University Zhejiang University Jinhua 321099 China; ^5^ Shaoxing Institute of Zhejiang University Zhejiang University Shaoxing 312000 China; ^6^ International Joint Innovation Center The Electromagnetics Academy at Zhejiang University Zhejiang University Haining 314400 China

**Keywords:** Cherenkov radiation, Water waves

## Abstract

Cherenkov radiation (CR) is a fascinating phenomenon that occurs not only in electromagnetic (EM) waves but also in water waves. The V‐shaped wake formed by a moving object on the water surface results from the constructive interference of water waves of different wavelengths, similar to CR. We designed and fabricated a one‐dimensional (1D) water wave crystal to analogize the behavior of moving particles in water waves. This crystal exhibits a band structure with both positive (positive group velocity) and negative slopes (negative group velocity), exciting and directing the water waves to produce forward and backward radiating wakes through a mechanism similar to CR. Notably, the radiation angle is easily adjustable. This method of regulating water wave wakes holds potential applications in scattering cancellation‐based wake stealth, navigation technology, and fluid dynamics for future offshore vessels.

## Graphical abstract

Water wave crystal analogizes the behavior of moving particles in water waves, creating forward and backward Cherenkov‐like radiating wake through positive and negative group velocities. By adjusting the ratio of wavelength to period, it is possible to modulate the radiation angle, which has potential applications in wake stealth, navigation technology, and fluid dynamics for offshore vessels.

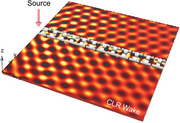

## 1 Introduction

When free electrons travel through a medium at a velocity greater than the phase velocity of light in that medium, a unique EM radiation phenomenon known as CR occurs.^[^ ^1^, ^2^, ^3^, ^4^, ^5^, ^6^, ^7^, ^8^, ^9^ ^]^ CR can be considered as an EM shock wave in the medium. This phenomenon can be observed in almost all classical wave systems, such as the acoustic shock wave produced by a supersonic object moving through the air. When an object, such as a boat or a duck, moves over calm water, it creates a fascinating feathery V‐shaped Kelvin wake behind it.^[^ ^10^, ^11^ ^]^ Water waves are highly dispersive and do not have a fixed speed (for shallow water waves, which have a speed $c=\sqrt{gh}$ that is related to the depth of water *h* and the acceleration of gravity g). Essentially, the Kelvin wake is a shock wave generated by water waves of different wavelengths, forming a wake through constructive interference. The study of wake currents is significant not only in physical research but also in navigation and hydrodynamics, with important practical implications for ship drag and bank erosion in navigable waterways.

The wake pattern of water waves was first explained by Lord Kelvin,^[^ ^12^ ^]^ who determined that the angle between the trajectory of a moving object and the wake half‐angle θ=arcsin(1/3)≈19.47 °. It was later demonstrated that this angle is related to the hull Froude number *Fr*, the velocity *c* of the water wave, the velocity *U* of the boat, among other factors. Despite these variables, the angle is usually considered to be fixed within a certain range. Additionally, combining Cherenkov physics and metamaterials has shown that when a medium has a negative refractive index or a negative group velocity dispersion, it emits a backward CR (the angle of radiation *θ* becomes obtuse), a property that effectively separates the emitted photons from the fast‐moving charges.^[^ ^7^, ^13^, ^14^, ^15^, ^16^ ^]^ The concept of backward propagating wake may offer new insights into scattering cancellation‐based wake stealth for ships. However, there is currently no conclusive evidence to support the existence of backward‐propagating wake in water wave systems.

When particles propagate within or near periodic structures (such as photonic crystals), unusual CR can occur. For instance, previous work proposed that a phased dipole array can precisely analogize moving charged particles to achieve backward CR.^[^ ^13^ ^]^ A subsequent study demonstrated that a 1D array of nanostructures can excite and guide surface plasmon wakes through a mechanism similar to CR.^[^ ^7^ ^]^ By leveraging the similarities between the two‐dimensional Maxwell equations and the water wave equations, we can apply methods used to control EM waves to the manipulation of water waves. Recent advancements have utilized these artificially designed structures to achieve phenomena such as superscattering,^[^ ^17^, ^18^ ^]^ cloaking^[^ ^19^, ^20^, ^21^, ^22^, ^23^ ^]^ and manipulation.^[^ ^24^, ^25^, ^26^, ^27^, ^28^, ^29^, ^30^ ^]^ Notably, two recent studies have utilized periodic media (or water wave crystals) to achieve effective negative water depth^[^ ^31^ ^]^ and hyperbolic dispersion^[^ ^32^ ^]^ without altering the water depth. This naturally leads to the question of whether it is possible to borrow electromagnetic wave methods to realize forward propagation or even backward propagation of the wake in a water wave system.

In this letter, we introduce a 1D water wave crystal utilized as an analog to moving particles in water waves, thereby enabling Cherenkov‐like radial (CLR) wake modulation in water wave systems. It exhibits a positive group velocity in a certain frequency range, generating a forward CLR wake, and a negative group velocity in another frequency range, generating a backward CLR wake. Notably, by adjusting the structural parameters and the incident wavelength, we can easily modify the radiation angle.^[^ ^33^ ^]^

## 2 Results

There are various types of water waves in nature, and this discussion focuses on surface gravity waves, which propagate along the interface between air and water, with gravity as the principal restoring force. We consider an inviscid, incompressible fluid and irrotational motion. The *x*‐*y* plane is set in the horizontal plane and *z*‐axis as the vertical axis, with the water depth denoted by *h*. The free surface of the calm water is at *z* = 0. Based on the linear theory of liquid surface waves, the velocity potential of the water surface Φ is

(1) Φx,y,z,t=Reφx,ycoshkz+he−iωt

Φ satisfies the 3D Laplace's equation ∇^2^Φ = 0, which is subjected to boundary conditions $\frac{\partial }{\partial z}\Phi =\frac{\omega^{2}}{g}\Phi$ on *z* = 0 and $\frac{\partial }{\partial n}\Phi =0$ on the interfaces between water and submerged, fixed rigid bodies, and n is the unit vector normal to interfaces. Where *ω*, g, *t* represent the angular frequency, the acceleration of gravity, and time. Equation (1) is a 3D problem whose numerical simulation is usually very time‐consuming, so it is usually approximated by simplifying it to a 2D problem in the *x‐y* plane. The reduced potential φ satisfies the 2D Helmholtz equation ∇^2^ *φ* + *k* ^2^ *φ* = 0. The dispersion relationship of water wave is *ω* ^*2*^ = gktanh(*kh*), *k* = 2π/*λ* represent the wave number. The vertical displacement of the liquid surface *η* is related to *φ* by η(x,y,t)=Re[−iωgφ(x,y)e−iωt]. The vertical displacement of the water‐air interface *η* satisfies the 2D partial differential equation.

(2) $$\nabla \cdot \left(u\nabla \eta \right)+\frac{\omega^{2}}{g}\eta =0$$

in which *u* = tanh (*kh*)/*k*. Meanwhile, the Maxwell's equations can be mapped to the water wave equations by a simple comparison. The Maxwell's equations for TM waves could be rewritten as $\nabla \cdot \left(\frac{1}{\varepsilon }\nabla H_{z}\right)+\frac{\omega^{2}}{c^{2}}\;H_{z}=0$, where *c* is the speed of light in vacuum, and ε is the dielectric constant. *H_z_* is the amplitude of the magnetic field in the z direction that can be corresponding to vertical displacement η. The phase velocity of water waves is $c_{p}=\frac{\omega }{k}=\sqrt{\frac{g}{k}\tanh\left(\mathrm{kh}\right)}$, and the group velocity is $\;c_{g}=\frac{d\omega }{dk}=\frac{1}{2}\;c_{p}\left(1+\frac{2kh}{\sinh(2kh)}\right)$.

**Figure 1:**
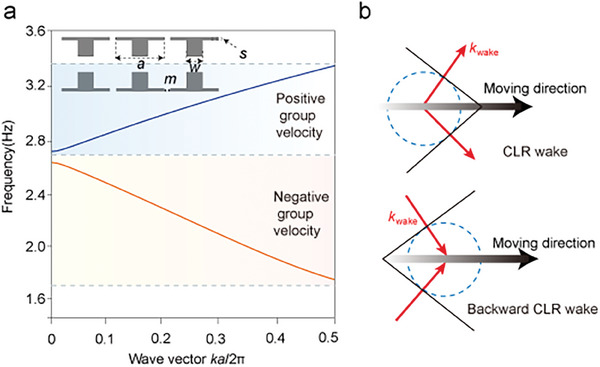
Band structure and CLR wake analysis in water wave crystals. a) Band structure of water wave crystals. The inset provides a schematic of the water wave crystal structure, with dimensions indicated. The blue region represents positive group velocity, while the orange region represents negative group velocity. b) Schematic of CLR wake. The red arrows indicate the *k_wake_*.

In the water with a periodic array, the eigenmodes of water waves are Bloch waves.^[^ ^34^, ^35^ ^]^ The dispersion relationship of Bloch waves can be accurately solved by using COMSOL Multiphysics with the 3D finite‐element method. As shown in **Figure** **1**a, we depict the band structure along the boundary of the irreducible Brillouin zone corresponding to the geometry outlined in the inset of Figure  1a, with a period of *a* = 8.9 cm. The spacing and positions of the structure are also designed as *m* = (2/15) *a* = 1.18 cm, *w* = (5/15) *a* = 2.97 cm, *s* = (1/15) *a* = 0.59 cm. By carefully designing the periodic structure of water wave crystals, the band structure and dispersion will exhibit regions with positive and negative slopes. Positive slopes denote equivalent positive group velocities within the crystals, while negative slopes denote equivalent negative group velocities. The gap *m* between the water wave crystals is small compared to the wavelength *λ*. Water waves incident within the crystals undergo Bragg scattering and radiate at an angle θ along the gap.

The angle of radiation is related to the ratio of *a*/*λ*. When the ratio is about 1 (*a*/*λ* ≈ 1), the wavelength is comparable to the period *a*, resulting in an acute angle of radiation. As shown in Figure  1b, the red arrows indicate the direction of the emitted wave. When *a*/*λ* ≈ 0, the wavelength is greater than the period, and the radiation angle becomes obtuse. The angle of the forward/backward CLR wake can be obtained by phase matching, i.e., matching the wave vector *k* inside the water wave crystal with the x‐direction component of the wake wave vector radiating outward (*k* = *k_wake_* sin*γ*). See Note S1 (Supporting Information).

**Figure 2:**
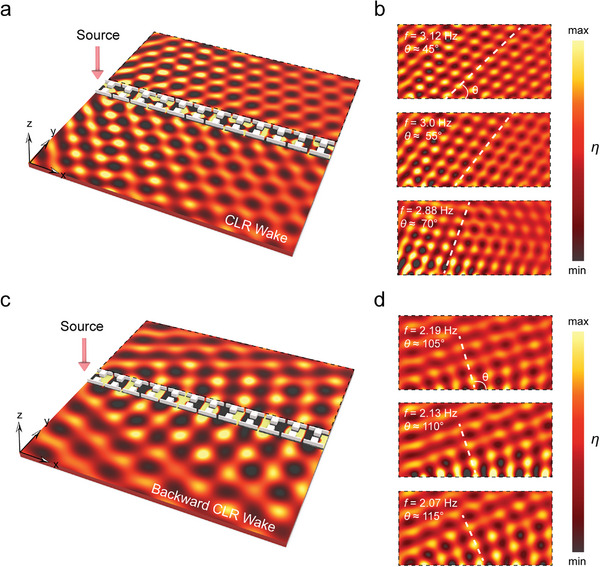
Schematic diagram of forward and backward CLR wake. a,b) Forward CLR wake with a source on the left for excitation, radiating outward after passing through a water wave crystal at an acute angle. c,d) Backward CLR wake, radiating outward after passing through a water wave crystal at an obtuse angle.

We performed numerical simulations of water wave CLR wake by solving Equation (2) using the PDE interface to simulate water waves. The simulation domain, measuring 1.2 m by 1.2 m, was bounded by an absorbing layer to prevent reflections. The water depth is *h* = 1 cm. We set up a source in the head of the structure, as shown in **Figure**  **2**a. The crystal structure exhibited positive group velocity and excited CLR wake when the frequency ranged from 2.8 to 3.3 Hz. As shown in Figure  2b, by changing the frequency, the radiation angle θ changed gradually: at *f* = 3.12 Hz, the angle was 45°; at *f* = 3.0 Hz, the angle was 55°; and at *f* = 2.88 Hz, the radiation angle was 70°. Similarly, as shown in Figure  2c, when the frequency ranged from 1.9 to 2.7 Hz, the crystal structure exhibited a negative group velocity and excited backward CLR wake. As shown in Figure  2d, by changing the frequency, the radiation angle *θ* also changes gradually: at *f* = 2.19 Hz, the angle was 105°; at *f* = 2.13 Hz, the angle was 110°; and at *f* = 2.07 Hz, the radiation angle was 115°. Extreme cases at the edges of the frequency range, where the radiation angle is either too large or too small to be accurately observed, are excluded. We also designed a smaller simulation of the water wave crystal structure. The simulation domain, measuring 0.25 m × 0.25 m  (The experimental domain, measuring 0.3 m × 0.3 m) was used for auxiliary validation. The detailed configuration can be found in Note S2 (Supporting Information).

## 3 Experimental Qualitative Verifications

**Figure 3:**
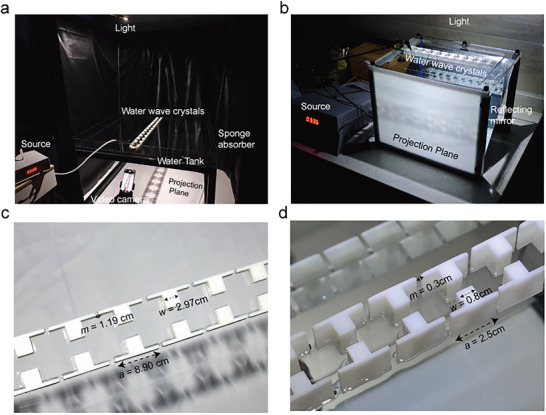
Experimental configuration of two different sizes of experimental wave tank. a) The wave tank is constructed from PMMA with dimensions of 1.2 m × 1.2 m. To reduce unnecessary wave reflections, the boundaries of the wave channel are lined with sponge wave‐absorbing panels. b) A small‐size wave tank with dimensions of 0.3 m × 0.3 m. The perimeter of the wave tank is curved to minimize unnecessary reflections. c,d) Water wave crystal structures of two different sizes were fabricated using 3D printing. A constant point light source is set up above, and a camera is placed below the tank to record the wave patterns. The point source of the water waves is connected to the wave generator via a conduit.

Two different sizes of experimental platforms were constructed. The larger experimental setup, shown in **Figure** **3**a, consists of a 1.2 m × 1.2 m PMMA tank. The water wave crystals were placed in the center of the tank and surrounded by a layer of wave‐absorbing sponges to minimize reflections from the tank boundaries. The smaller experimental setup, shown in Figure  3b, consists of a 0.3 m × 0.3 m glass tank. Separate constant light sources above the tank provide illumination, projecting waves onto a white projection cloth below the tank (larger platforms) or reflecting them through mirrors onto a flat projection board (smaller platforms) to improve visibility. Cameras placed below or to the side capture these projections.

Figure  3c shows a water wave crystal fabricated using a 3D printer. The height of the structure is 2 cm. The smaller water wave crystal shown in Figure  3d has a lattice constant of *a* = 2.5 cm, *m* = 0.33 cm, *w* = 0.83 cm, *s* = 0.17 cm. The water depth *h* = 0.5 cm. The excitation of water waves is achieved by a pneumatic source. Specifically, the point source for the larger water wave crystals consists of an air tube with an inner diameter of 0.5 cm and an outer diameter of 0.8 cm, while for the smaller water wave crystals, the aforementioned air tube is connected to a nozzle with an outer diameter of 0.2 cm.

**Figure 4:**
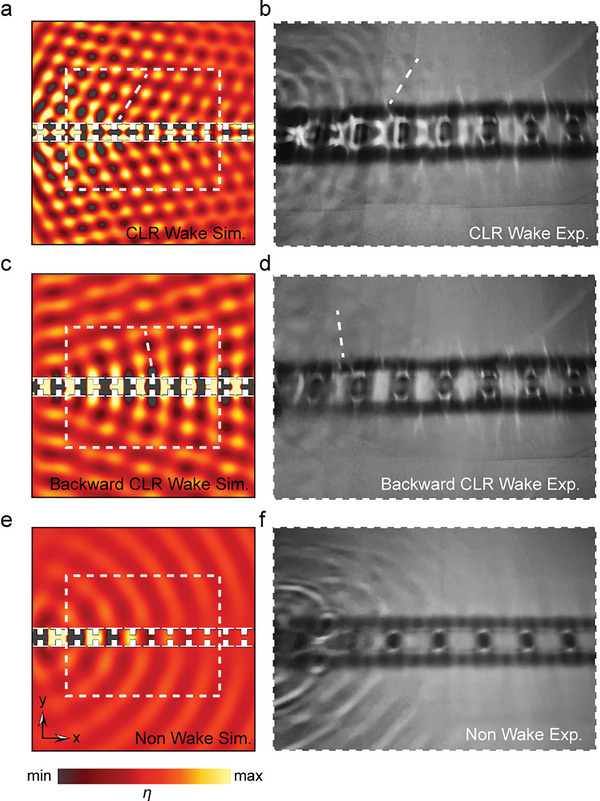
Simulation and experimental comparison for verifying the forward/backward CLR wake of water waves. The figures on the left illustrate field maps derived from simulations, with black arrows marking the propagation direction. Our projection area is the area marked with a white dotted line, which is about 1 m × 0.8 m, and the white dotted line on the left is the same size as the experimentally validated area photographed on the right. a,b) are comparisons of simulations and experimental plots of forward CLR wake of water waves. c,d) are comparisons of simulations and experimental plots of backward CLR wake of water waves. e,f) are comparisons of simulations and experimental plots of Non CLR wake.

As shown in **Figure** **4**a,b, we selected a frequency of 2.95 Hz to illustrate that the water wave passing through the water wave crystal radiates as a forward CLR wake. The radiation angle observed in the experimental photographs is about 60°, which aligns well with the simulation results. Since the incident amplitude of the water wave is small (satisfying the condition of linear wave, allowing the nonlinearity to be neglected) and the water wave loss due to the structure is relatively large, the water wave can only be transmitted to half the length of the structure. However, this does not affect our conclusions. As the incident amplitude gradually increases, the water wave begins to break up, and the nonlinear effects become increasingly significant. We ultimately determined a suitable incident amplitude.

Afterward, we adjusted the frequency to 2.13 Hz to demonstrate the backward CLR wake, as shown in Figure  4c,d, which captures the radiation at an angle of 110°. The experimentally derived images are consistent with the simulation results. The backward CLR wake requires further amplitude reduction, so we provided more fine‐grained control over the amplitude. Additionally, we also presented the patterns without any radiation, as shown in Figure  4e,f. We recommend Video S1 (Supporting Information) for a better demonstration. It is worth noting that in experiments with larger water wave crystals, the angular variation of both forward and backward CLR wakes is visually indistinguishable. To further investigate the mechanism of wake control, we carried out an auxiliary validation using a small experimental setup. Due to the small wave amplitude, the screenshots are not clear; please refer to Note S1 and Video S2 (Supporting Information). The radiation angle can be varied within a certain range using the small water wave crystal experiment. The water waves attenuate after passing through a certain number of crystals due to fluid‐solid interaction. Based on the experimental results, fewer cells lead to lower attenuation of water waves within the structure and a broader range of angular variation, which is in better agreement with the theoretical predictions. However, it primarily serves as additional confirmation rather than a substantial contribution due to the smaller amplitude.

## 4 Conclusion

In summary, we successfully designed and fabricated two different sizes of 1D water wave crystals, using them as analogs to moving particles in water waves, thereby enabling CLR wake modulation in water wave systems. By achieving positive and negative group velocities within the crystals, we realized both forward and backward CLR wakes. Notably, by adjusting the incident wavelength, we can easily modify the radiation angle. Both experimental and simulation results demonstrate the effectiveness of the water wave crystals. Our approach is valuable for controlling water wave radiation and deepening our understanding of water wave propagation mechanisms. This could potentially provide new insights for wake stealth in ships, maritime technology, and fluid dynamics in the future.

## Conflict of Interest

The authors declare no conflict of interest.

## Author Contributions

Y.Y., H.C., and H.W. created the design. H.C. designed the experiment. Y.Y. carried out the measurement with the assistance from Z.Q.; L.S. analyzed the data. Z.Q. performed simulations, H.W. and H.C. provided the theoretical explanations. Z.Q. wrote the manuscript with the input from L.S and H.C. H.W. supervised the project. All authors contributed extensively to this work.

## Supporting information



Supporting Information

Supplementary Video 1

Supplementary Video 2

## Data Availability

The data that support the findings of this study are available in the supplementary material of this article.

## References

[1] I. Carusotto , M. Artoni , G. C. La Rocca , F. Bassani , Phys. Rev. Lett. 2001, 87, 064801. 11497831 10.1103/PhysRevLett.87.064801

[2] Y. Adiv , H. Hu , S. Tsesses , R. Dahan , K. Wang , Y. Kurman , A. Gorlach , H. Chen , X. Lin , G. Bartal , I. Kaminer , Phys. Rev. X 2023, 13, 001002.

[3] H. Hu , X. Lin , L. J. Wong , Q. Yang , D. Liu , B. Zhang , Y. Luo , eLight 2022, 2, 1.

[4] X. Lin , S. Easo , Y. Shen , H. Chen , B. Zhang , J. D. Joannopoulos , M. Soljačić , I. Kaminer , Nat. Phys. 2018, 14, 816.

[5] S. N. Galyamin , A. V. Tyukhtin , Phys. Rev. Lett. 2014, 113, 064802. 25148331 10.1103/PhysRevLett.113.064802

[6] V. Ginis , J. Danckaert , I. Veretennicoff , P. Tassin , Phys. Rev. Lett. 2014, 113, 167402. 25361279 10.1103/PhysRevLett.113.167402

[7] P. Genevet , D. Wintz , A. Ambrosio , A. She , R. Blanchard , F. Capasso , Nat. Nanotechnol. 2015, 10, 804. 26149237 10.1038/nnano.2015.137

[8] H. Hu , X. Lin , D. Liu , H. Chen , B. Zhang , Y. Luo , Adv. Sci. 2022, 9, 2200538. 10.1002/advs.202200538 PMC9475543 35863914

[9] Z. Su , B. Xiong , Y. Xu , Z. Cai , J. Yin , R. Peng , Y. Liu , Adv. Opt. Mater. 2019, 7, 1801666.

[10] F. Moisy , M. Rabaud , Phys. Rev. E 2014, 90, 023009. 10.1103/PhysRevE.90.023009 25215822

[11] M. Rabaud , F. Moisy , Phys. Rev. Lett. 2013, 110, 214503. 23745883 10.1103/PhysRevLett.110.214503

[12] W. I. Thomson , Proc. R. Soc. London 1887, 42, 80.

[13] S. Xi , H. Chen , T. Jiang , L. Ran , J. Huangfu , B.‐I. Wu , J. A. Kong , M. Chen , Phys. Rev. Lett. 2009, 103, 194801. 20365927 10.1103/PhysRevLett.103.194801

[14] X. Guo , C. Wu , S. Zhang , D. Hu , S. Zhang , Q. Jiang , X. Dai , Y. Duan , X. Yang , Z. Sun , S. Zhang , H. Xu , Q. Dai , Nat. Commun. 2023, 14, 2532. 37137873 10.1038/s41467-023-37923-w PMC10156754

[15] X. Lin , H. Hu , S. Easo , Y. Yang , Y. Shen , K. Yin , M. P. Blago , I. Kaminer , B. Zhang , H. Chen , J. Joannopoulos , M. Soljacic , Y. Luo , Nat. Commun. 2021, 12, 5554. 34548482 10.1038/s41467-021-25822-x PMC8455627

[16] Z. Duan , X. Tang , Z. Wang , Y. Zhang , X. Chen , M. Chen , Y. Gong , Nat. Commun. 2017, 8, 14901. 28332487 10.1038/ncomms14901 PMC5376646

[17] X. Liang , Z. Zhang , J. Chu , J. Luo , D. Meng , Z. Zhou , Mater. Today Commun. 2024, 38, 107755.

[18] Z. Qin , C. Qian , L. Shen , X. Wang , I. Kaminer , H. Chen , H. Wang , Natl. Sci. Rev. 2023, 10, nwac255. 37266547 10.1093/nsr/nwac255 PMC10232047

[19] F. Tay , Y. Zhang , H. Xu , H. Goh , Y. Luo , B. Zhang , Natl. Sci. Rev. 2021, 205, 6430182.

[20] S. Zou , Y. Xu , R. Zatianina , C. Li , X. Liang , L. Zhu , Y. Zhang , G. Liu , Q. H. Liu , H. Chen , Z. Wang , Phys. Rev. Lett. 2019, 123, 074501. 31491099 10.1103/PhysRevLett.123.074501

[21] J. Park , J. R. Youn , Y. S. Song , Phys. Rev. Lett. 2019, 123, 074502. 31491109 10.1103/PhysRevLett.123.074502

[22] Y. Hua , C. Qian , H. Chen , H. Wang , Mater. Today Phys. 2022, 27, 100754.

[23] T. Iida , A. Zareei , M.‐R. Alam , J. Fluid Mech. 2022, 954, A4.

[24] S. Zhu , X. Zhao , L. Han , J. Zi , X. Hu , H. Chen , Nat. Rev. Phys. 2024, 6, 231.

[25] Z. Che , W. Liu , J. Ye , L. Shi , C. T. Chan , J. Zi , Phys. Rev. Lett. 2024, 132, 044001. 38335365 10.1103/PhysRevLett.132.044001

[26] X. Zhao , X. Hu , J. Zi , Phys. Rev. Lett. 2021, 127, 254501. 35029421 10.1103/PhysRevLett.127.254501

[27] Y. Meng , Y. Hao , S. Guenneau , S. Wang , J. Li , New J. Phys. 2021, 23, 073004.

[28] F. De Vita , F. De Lillo , F. Bosia , M. Onorato , Phys. Fluids 2021, 33, 077122.

[29] N. Laforge , V. Laude , F. Chollet , A. Khelif , M. Kadic , Y. Guo , R. Fleury , New J. Phys. 2019, 21, 083031.

[30] C. Li , L. Xu , L. Zhu , S. Zou , H. Liu , Z. Wang , H. Chen , Phys. Rev. Lett. 2018, 121, 104501. 30240256 10.1103/PhysRevLett.121.104501

[31] L. Han , S. Chen , H. Chen , Phys. Rev. Lett. 2022, 128, 204501. 35657890 10.1103/PhysRevLett.128.204501

[32] L.‐P. Euvé , K. Pham , A. Maurel , J. Fluid Mech. 2023, 961, A16.

[33] J. Scruggs , P. Jacob , Science 2009, 323, 1176. 19251617 10.1126/science.1168245

[34] X. Hu , C. T. Chan , K. M. Ho , J. Zi , Phys. Rev. Lett. 2011, 106, 174501. 21635037 10.1103/PhysRevLett.106.174501

[35] X. Hu , C. T. Chan , Phys. Rev. Lett. 2005, 95, 154501. 16241730 10.1103/PhysRevLett.95.154501

