# Supplementary material for: Observations of Cherenkov‐Like Radial Wake in Water Waves
Source: Adv Sci (Weinh). 2025 Jan 28;12(11):2412638. doi: 10.1002/advs.202412638 (PMC11923993; doi:10.1002/advs.202412638)
Supplement: Supplementary file 1 — Supporting Information [file ADVS-12-2412638-s003.docx]

**Supplementary** **Information for**

Observations of Cherenkov-like radial wake in water waves

Supplementary Note 1: Phase matching


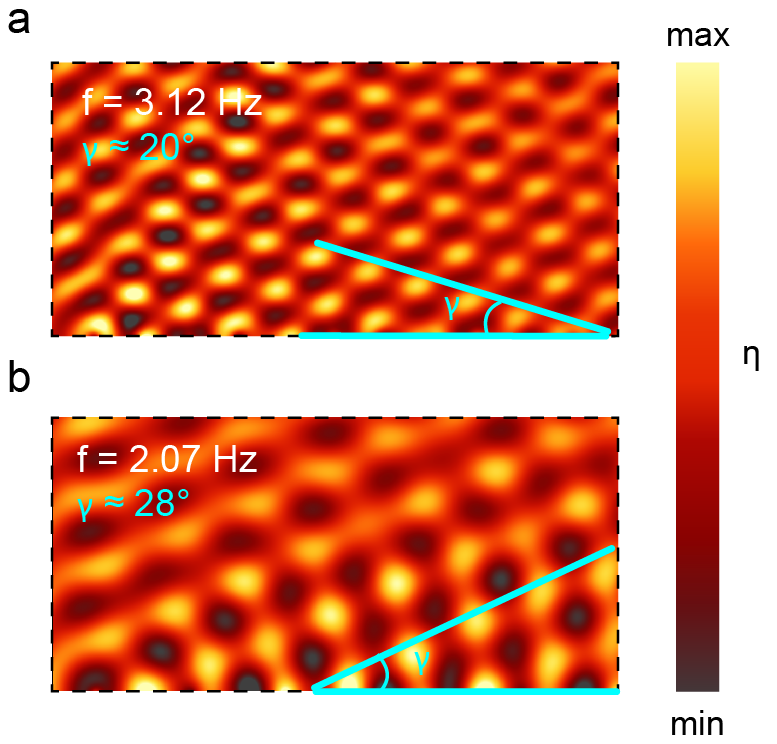


Figure S1. Forward/ Backward CLR Wake Schematic. This figure is part of Figures 2b and 2d to show that the angle $\gamma$. Both forward- and backward-propagating CLR wake are taken at an acute angle.

The angle of the forward/backward CLR wake can be determined by phase matching, i.e., matching the wave vector $k$ inside the water wave crystal with the x-direction component of the wake wave vector radiating outward ($k=k_{wake}\sin\gamma$, $k_{wake} ={2\pi}/\lambda$), $\gamma$is defined as the angle between the wavefront surface and the x direction.

Supplementary Note 2: Simulation of small water wave crystal

The simulation domain, measuring 0.25 m by 0.25 m, was bounded by an absorbing layer to prevent reflections. The water depth is$h = 0.5 \mathrm{cm}$. As shown in Figure S1a-S1c, by changing the frequency, the radiation angle θ changed gradually: at $f$ = 6.98 $\mathrm{Hz}$, the angle was 40°; at $f$ = 6.79 $\mathrm{Hz}$, the angle was 45°; and at $f$ = 6.58 $\mathrm{Hz}$, the radiation angle was 60°.

Similarly, as shown in Figure S1b-S1f, altering the frequency led to gradual changes in the radiation angle θ: at $f$ = 5.27 $\mathrm{Hz}$, the angle was 95°; at $f$ = 5.11 $\mathrm{Hz}$, the angle was 105°; and at $f$ = 4.93 $\mathrm{Hz}$, the radiation angle was 110°. Extreme cases at the edges of the frequency range, where the radiation angle is either too large or too small to be accurately observed, are excluded.


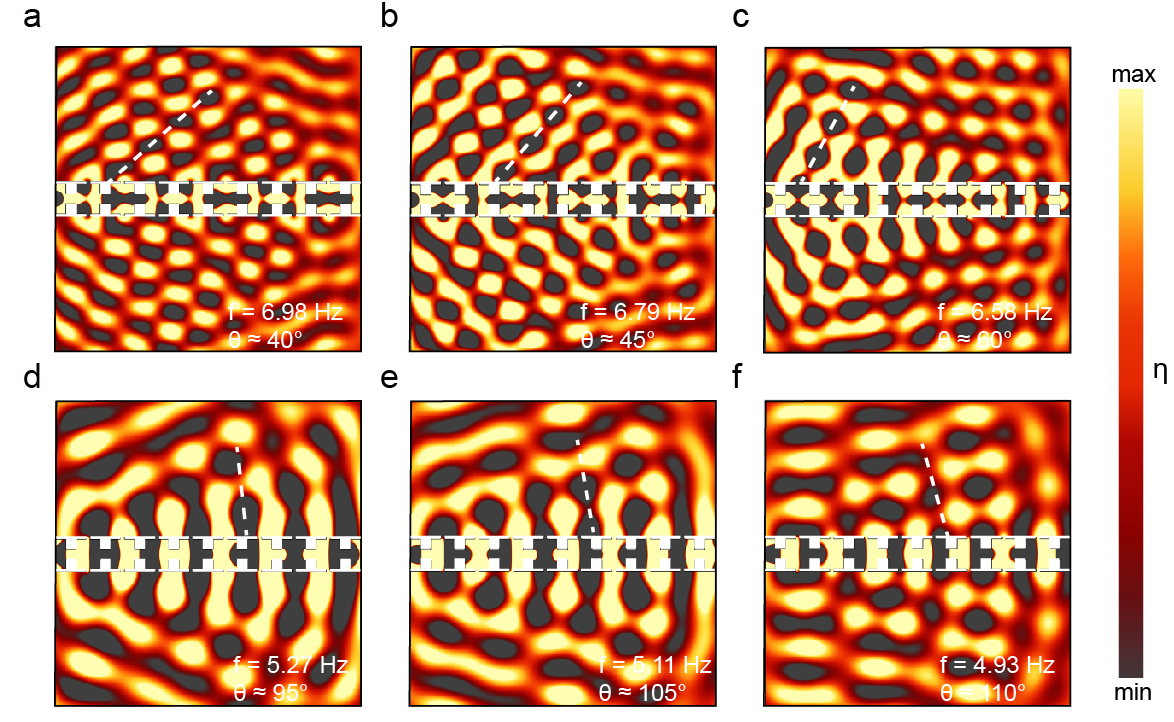


Figure S2. Forward/ Backward CLR Wake Schematic of a Small Water Wave Crystal.

Since the wave amplitude is small in the experiment, the image directly taken from the screenshot is insufficient in terms of clarity. Observing the change in the wake angle is better facilitated through the experimental video rather than relying solely on static images. Figure S2 serves a demonstrative purpose, displaying a single frame extracted from the video capturing a small water wave crystal. The angle of the wake is represented by the angle between the black dashed line and the white dashed line. The left three panels illustrate the forward wake, whereas the right three panels depict the backward wake.


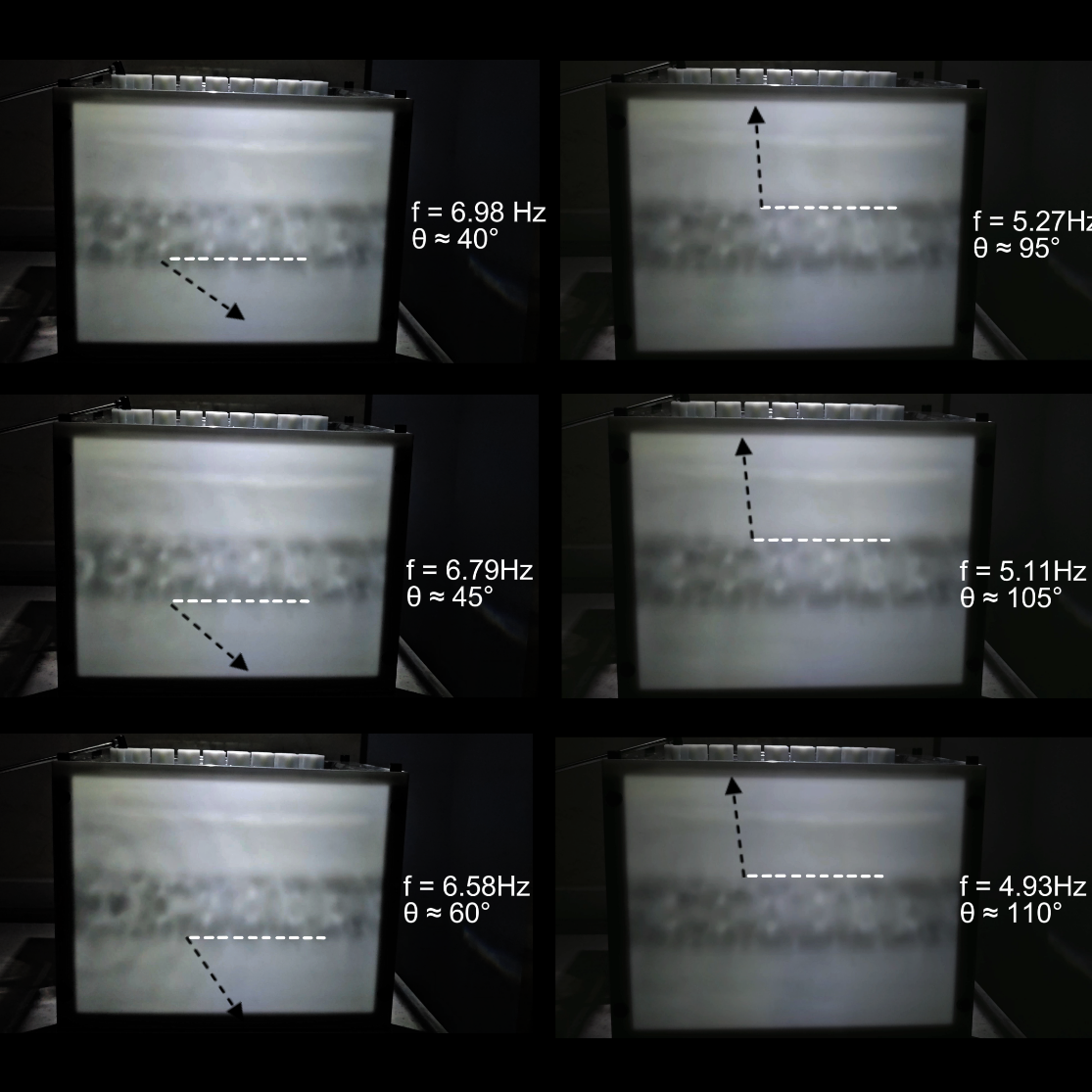


Figure S3. Screenshot of Forward/Backward CLR Wake Experiment Video for Small Water Wave Crystals.

Supplementary Note 3: Supplementary video description

- Supplementary Video S1 (.mp4 format). Experimental video of larger water wave crystals verifying forward/backward CLR wake.
- Supplementary Video S2 (.mp4 format). Smaller water wave crystals experimentally verified with angle change.
